# Fascia Iliaca Blocks: A Cadaveric Study Comparing the Suprainguinal Approach to the Loss of Resistance Technique

**DOI:** 10.7759/cureus.38243

**Published:** 2023-04-28

**Authors:** Diego A Abelleyra Lastoria, Zuzanna Halicka, Kin C Liu, Elsie Osei Bonsu, Mark Reaveley, David Parry

**Affiliations:** 1 Faculty of Life Sciences and Medicine, King's College London, London, GBR; 2 Department of Anesthesiology, Frimley Health NHS Foundation Trust, London, GBR

**Keywords:** suprainguinal approach, neck of femur fracture, loss of resistance technique, fascia iliaca blocks, cadaveric study

## Abstract

Background

Local anesthetic fascia iliaca blocks (FIB) are used for peri- and post-operative analgesia in hip fracture patients. The loss of resistance technique (LORT) and the suprainguinal approach (SIA) are two techniques commonly used. We present a pilot, first cadaveric study model that compares both techniques.

Methods

Methylene blue dye was injected as a local anesthetic substitute. This dye is easily visible. Both hips on each of the seven cadavers were injected with one of the two techniques used per side. Single-blinded randomization was conducted to determine the technique to be administered in each hip. Ten minutes after injection, the dissection of the femoral, obturator, and lateral femoral cutaneous nerves (LFCN) revealed the dye spread around these nerves. The SIA and the LORT were compared in their area of dye distribution using the Wilcoxon matched-pairs signed-rank test. The hips of a single cadaver were matched, since these received the injection via a different technique.

Results

The area of dye spread was greater in the SIA for five cadavers. Dye spread was greater following the LORT in two cadavers. However, the Wilcoxon matched-pairs signed-ranked test revealed no statistically significant difference in the area of dye spread following both techniques (p= 0.866). The SIA showed that the femoral, obturator, and LFCN were stained in six, three, and seven cadavers, respectively. The LORT resulted in five, two, and five of these nerves being stained, respectively.

Conclusion

This study found no statistically significant difference in terms of the area of dye spread between the SIA and the LORT. The number of nerves stained was greater following the SIA. This suggests that the SIA may lead to superior anesthetic outcomes. This conclusion is limited by the low sample size in this study. This work warrants the collection of more data through the same method to support or challenge our findings.

## Introduction

Neck of femur fractures have an incidence of 70,000-75,000 cases per year in the United Kingdom [1]. They have a 10% mortality within one month and 30% within one year. Hip fractures collectively lead to medical and social care costs of approximately £2 billion [1]. Adequate analgesia is key for reducing morbidities. Higher pain scores lead to longer hospital stays, a decreased likelihood of ambulating post-operatively, lower locomotion scores, and long-term functional impairment [2]. In addition, untreated pain following hip fractures in cognitively normal adults leads to an increased risk of delirium [2]. Patients with moderate/severe pain following a neck of femur fracture can experience increased difficulty when performing activities of daily living and worsened quality of life than patients with no or mild pain [3].

Treatment guidelines recommend paracetamol and opioids to manage pain in patients with a neck of femur fracture [4]. Opioid side effects include arrhythmias, constipation, hallucinations, nausea, and palpitations [5]. Opioids can lead to addiction and withdrawal in the long term. Treatment guidelines recommend nerve blocks if opioids and paracetamol do not work or to limit opioid usage [4]. The femoral, obturator, and lateral femoral cutaneous nerves (LFCN) must be blocked to achieve analgesia of the hip joint. Anesthetizing these nerves with a local anesthetic leads to sensory blockade. Motor blockade is also achieved, resulting in decreased muscle tension and bone impaction, reducing pain.

Fascia iliaca blocks (FIB) are used to anesthetize the hip and thigh. They involve injecting local anesthetic into the fascial plane between the fascia iliaca and its underlying iliacus muscle [6]. Local anesthetic spreads within this fascial plane to reach the target nerves. The loss of resistance technique (LORT) and the suprainguinal approach (SIA) are two FIB techniques commonly used in clinical practice. The LORT was first described by Dalens et al. [7]. A needle is inserted perpendicular to the skin. A first loss of resistance is felt as the needle perforates the fascia lata, with a subsequent one felt through the fascia iliaca. The local anesthetic is delivered inferior to the inguinal ligament. The SIA (developed by Hebbard et al. [6]) involves advancing a needle deep into the inguinal ligament to inject local anesthetic beneath the fascia iliaca. This is delivered superior to the inguinal ligament. This technique relies on ultrasound visualization to guide the needle to the correct location.

No studies comparing the SIA and the LORT were identified in the literature. We aimed to compare the efficacy of the SIA and the LORT by comparing dye spread (local anesthetic substitute) and nerves stained in each technique following bilateral injections in seven fresh frozen cadavers.

## Materials and methods

Institutional review board approval was not required due to this being a cadaveric study. All ethical requirements outlined in the Human Tissue Act (2004) regarding the use of cadavers in research were followed. The dissection was conducted at the King's College London anatomy department. Seven un-embalmed fresh frozen cadavers received bilateral injections of methylene blue dye. A total of 30 mL were injected into each hip by an experienced anesthetist. This was a mixture of 27 mL normal saline and 3 mL methylene blue dye. Single-blinded randomization was conducted to determine the injection administered in each hip. Both hips of a single cadaver received a different technique. This allowed the reliable comparison of dye spread resulting from both techniques (assuming there are no significant differences in anatomy between the hip joints of a single cadaver). A Nikon high-definition digital camera (Nikon Corporation, Tokyo, Japan) with automatic function was used throughout. Flash was enabled.

Injection of methylene blue dye via the suprainguinal approach

**Figure 1:**
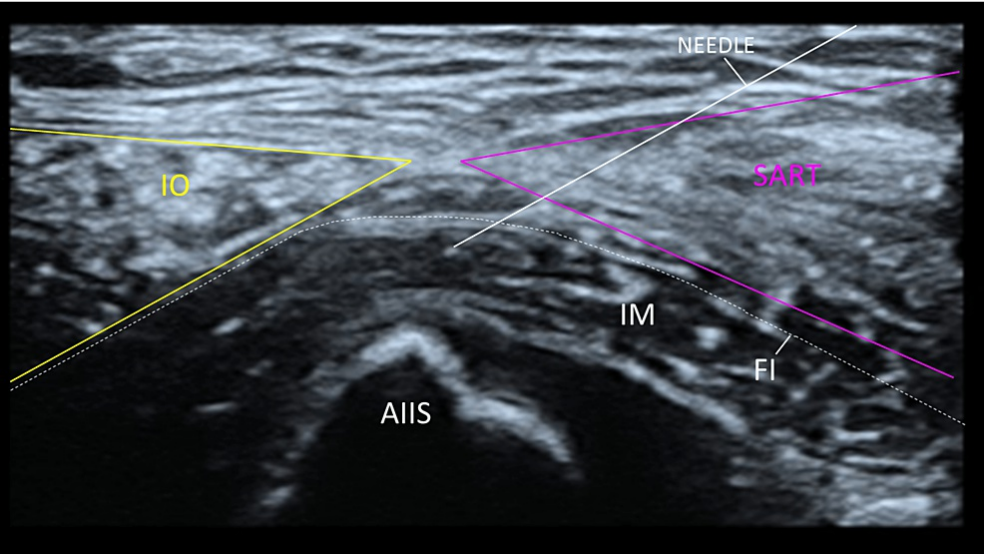
The hip as visualized under ultrasound guidance during the suprainguinal approach The "bow tie" appearance of the internal oblique (yellow) and sartorius (purple) muscles can be appreciated. A block needle, demonstrated by a white line, is placed inferior to the fascia iliaca IO, internal oblique muscle; SART, sartorius muscle; IM, iliacus muscle; AIIS, anterior inferior iliac spine; FI, fascia iliaca

A modified version of Hebbard et al.'s technique [6] was followed. The hip was placed in an extended position, with the cadaver lying supine. The injection was performed with a high-frequency linear ultrasound transducer (probe), imaging to 4 cm depth. A Sonosite SII ultrasound machine (Fujifilm Sonosite, Bothell, WA) was used. The probe was placed over the inguinal ligament, 5 cm medial to the anterior superior iliac spine (ASIS), and was orientated in the parasagittal plane. The ilium can be identified as a thick white line. The iliacus muscle can be identified, with the fascia iliaca covering its surface (Figure 1). To enhance the imaging of the fascia iliaca, the probe was tilted to orientate the beam perpendicular to the fascia.

The probe was moved inferomedially along the inguinal ligament until the femoral artery was imaged. The probe was then translated superolaterally along the inguinal ligament until the anterior inferior iliac spine was imaged. In this position, the probe was lateral to the femoral nerve. The deep circumflex iliac artery could then be identified superficial to the fascia iliaca, 1-2 cm superior to the inguinal ligament. This artery was used as a landmark for needle placement. A 80 mm-long 22 G PAJUNK SonoPlex (Baden-Württemberg, Germany) needle was introduced through the skin approximately 2-4 cm inferior to the inguinal ligament. The needle was inserted parallel to the probe, in plane with the ultrasound beam. The needle was advanced through the fascia iliaca at the level of the inguinal ligament. The tip of the needle was inserted 2-4 cm relative to the skin entry point. To verify adequate needle position, 1 mL of dye was injected, to confirm there is no fascial line between the injectate and the iliacus muscle. Upon observing this, the needle was advanced deep into the fascia iliaca and into the iliac fossa, moving into the space created by the distending fluid. The fascia iliaca should be observed separating the fluid from the deep circumflex iliac artery. A total of 29 mL of methylene blue dye was injected at this position (5 mL was injected followed by aspiration to mimic what the operator would do in clinical practice). Following aspiration, the remaining 24 mL was injected. Imaging was performed throughout to confirm that the dye was not being injected intravascularly. The ultrasound image was recorded by a separate anesthetist before and after injection to identify the number of fascial layers post hoc.

Injection of methylene blue dye via the loss of resistance technique

Ultrasound image was recorded by an anesthetist prior to and after injection. A modified version of Dalens et al.'s technique [7] was followed. The cadaver was placed in a supine position. A projection of the inguinal ligament was drawn on the skin. The line was divided in three equal parts. The puncture site was marked 0.5 cm caudal to the boundary between the lateral and middle thirds. A 50 mm-long 18 G blunt needle was inserted perpendicular to the skin while exerting gentle pressure on the barrel. A photographic record was kept. A "give," followed by a loss of resistance, was felt as the tip of the needle pierced the fascia lata. Upon advancing the needle further, another "give" followed by a loss of resistance was felt as the fascia iliaca was pierced. The first 5 mL of methylene blue dye was injected to check for any resistance to injection. Following aspiration, the remaining 25 mL was injected. Firm compression immediately caudal to the needle was applied at all times while injecting to favor the upward spread of the dye. After withdrawing the needle, the groin was firmly massaged to improve rostral diffusion of the dye within the fascia iliaca compartment.

Dissection technique following injection

Each cadaver was dissected by an experienced clinical anatomist 10 minutes post hoc. Standard, student issue cadaveric dissection equipment was used. Three incisions were performed bilaterally. The first one extended from the ASIS to the point halfway between the pubic symphysis and umbilicus. The second one began 3 cm inferior to the ASIS and terminated 3 cm inferior to the pubic symphysis. The third incision extended from the pubic symphysis to the point halfway between it and the umbilicus (i.e., the termination of the first incision).

The skin and abdominal wall were reflected laterally. The LFCN was identified deep into the inguinal ligament, close to its origin at the ASIS. The femoral nerve was identified deep into the midpoint of the inguinal ligament. Soft tissues superolateral to the pubic symphysis were dissected to identify the iliopectineal line of the pelvic girdle. This was traced inferomedially to identify the obturator foramen and the obturator nerve running through the obturator canal.

Photographs of the spread of dye were taken. The following were recorded: directional spread from puncture site (medial to lateral and superior to inferior, measured with a 1 cm grid), area of dye spread (calculated by multiplying medial-lateral and superior-inferior spread), losses of resistance (or "pops") felt during the LORT, nerves stained by the methylene blue dye, number of fascial layers identified under ultrasound after injection, and the quality of the ultrasound image in the SIA ("poor," "adequate," "good," or "excellent").

Statistical analysis

The SIA and the LORT were compared in their area of dye distribution, using the Wilcoxon matched-pairs signed-rank test. Data points had unequal variances and were not normally distributed, preventing the performance of the parametric paired t-test. The hips of a single cadaver were matched, since these received the injection via a different technique. The ability of the SIA and the LORT to stain the femoral, obturator, and LFCN was assessed by comparing the number of nerves stained following each technique. The performance of chi-squared test was prevented by the small sample size and because the LORT and SIA could not be tabulated as independent variables (since a nerve could be stained following both approaches). The critical level of statistical significance was set to 0.05 (5%). Statistical analysis was performed using Statistical Package for Social Sciences (SPSS) version 29.0 (IBM SPSS Statistics, Armonk, NY).

## Results

Seven cadavers received bilateral dye injections. Each hip received an injection via the SIA or the LORT. The directional spread of dye and the area of dye spread in each cadaver are presented in Tables 1-2.

**Table 1:** Directional spread of dye and the area of dye spread in seven cadavers following the loss of resistance technique

Cadaver	Cadaver 1	Cadaver 2	Cadaver 3	Cadaver 4	Cadaver 5	Cadaver 6	Cadaver 7
Superior-inferior spread	18 cm	16 cm	10 cm	8 cm	10 cm	9 cm	10 cm
Medial-lateral spread	13.5 cm	13.5 cm	8.5 cm	4 cm	5 cm	2 cm	5 cm
Area of spread	243 cm^2^	216 cm^2^	85 cm^2^	32 cm^2^	50 cm^2^	18 cm^2^	50 cm^2^

**Table 2:** Directional spread of dye and the area of dye spread in seven cadavers following the suprainguinal approach

Cadaver	Cadaver 1	Cadaver 2	Cadaver 3	Cadaver 4	Cadaver 5	Cadaver 6	Cadaver 7
Superior-inferior Spread	10 cm	7 cm	12 cm	15 cm	12 cm	9 cm	12 cm
Medial-lateral spread	10.5 cm	8 cm	8 cm	8 cm	5 cm	5 cm	6 cm
Area of spread	105 cm^2^	56 cm^2^	96 cm^2^	90 cm^2^	60 cm^2^	45 cm^2^	72 cm^2^

**Figure 2:**
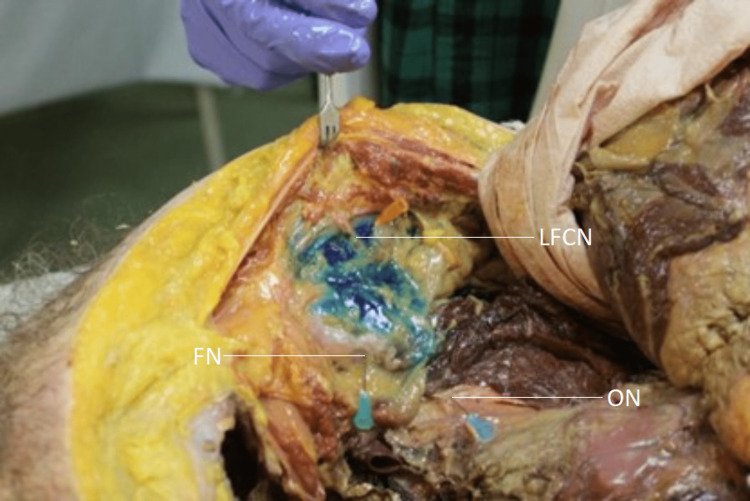
Nerves stained following the loss of resistance technique in cadaver 3 LFCN, lateral femoral cutaneous nerve; FN, femoral nerve; ON, obturator nerve

The area of dye spread was greater in the SIA for five cadavers. Dye spread was greater following the LORT in two cadavers. The Wilcoxon matched-pairs signed-ranked test revealed no statistically significant difference in the area of dye spread following the SIA and the LORT (p = 0.866). Table 3 depicts the nerves stained in each cadaver. Three cadavers (2, 3, and 5) had the same nerves stained bilaterally. Cadaver 3 had only the LFCN stained bilaterally (Figure 2).

**Table 3:** Nerves stained in each cadaver following the suprainguinal approach and the loss of resistance technique Yes, nerve stained; no, nerve unstained LORT, loss of resistance technique; SIA, suprainguinal approach; LFCN, lateral femoral cutaneous nerve

Nerve	Cadaver 1		Cadaver 2		Cadaver 3		Cadaver 4		Cadaver 5		Cadaver 6		Cadaver 7	
	LORT	SIA	LORT	SIA	LORT	SIA	LORT	SIA	LORT	SIA	LORT	SIA	LORT	SIA
Femoral	Yes	Yes	Yes	Yes	No	No	No	Yes	Yes	Yes	Yes	Yes	Yes	Yes
LFCN	Yes	Yes	Yes	Yes	Yes	Yes	No	Yes	Yes	Yes	No	Yes	Yes	Yes
Obturator	No	Yes	Yes	Yes	No	No	No	No	No	No	No	Yes	Yes	No

Dye was injected into the correct fascial plane for all cadavers, with the exception of the hip receiving the LORT in cadaver 4. This resulted in no nerves being stained in this hip. The femoral nerve was stained in five cadavers following the LORT, compared to six cadavers with the SIA. The LFCN was stained in five cadavers following the LORT, while the dye reached the LFCN in all cadavers with the SIA. The obturator nerve was stained in two cadavers following the LORT, compared to three cadavers with the SIA (Table 3).

Two "pops" (losses of resistance) were felt while performing the LORT in all cadavers. Ultrasound image quality during the SIA was deemed "excellent" for cadavers 3 and 7 and "good" for the remaining five cadavers. Two fascial layers were identified under ultrasound for all cadavers post injection.

## Discussion

Hebbard et al. conducted a cadaveric study to evaluate the SIA [6]. They performed bilateral injections of the dye in six un-embalmed cadavers. The femoral nerve was stained in all injections. The LFCN was stained bilaterally in all cadavers in which it was present (it was absent bilaterally in one cadaver). In the present study, the SIA stained the femoral nerve in six cadavers and the LFCN in all. In addition, Hebbard et al. performed over 150 blocks using this technique, without complications [6]. This attests to the safety of the SIA.

Dalens et al. administered the LORT in 60 pediatric patients undergoing lower limb surgery, including femur fractures, the removal of implants, and the repair of slipped capital femoral epiphyses [7]. Adequate analgesia was achieved in 90% of subjects. In addition, a motor blockade was observed, which led to decreased bone impaction and lower pain preoperatively. Yuan et al. administered a FIB using the LORT to 175 patients prepared for hip surgery [8]. They found that 160 patients (91.4%) experienced changes in temperature sensation after 20 minutes, and 119 experienced changes in temperature sensation in all areas supplied by the nerves assessed (femoral, obturator, and LFCN) (68% block rate). This is consistent with the findings by Dalens et al., who found that 90% of the patients who received a FIB via the LORT achieved adequate analgesia [7]. Their concordance strengthens the claim that the LORT is effective at providing anesthesia.

Current evidence suggests that the SIA and the LORT are effective techniques to administer FIB. To the best of our knowledge, this is the first cadaveric study comparing both techniques. Statistical analysis revealed no difference in the area of dye spread between the SIA and the LORT. The number of nerves stained was greater following the SIA. This suggests that the SIA may lead to superior anesthetic outcomes. In vivo comparisons of both techniques are required to ascertain the validity of this claim.

The choice of technique is likely to be influenced by factors other than anesthetic efficacy. Administering a FIB via the SIA requires proficiency in nerve blocks under ultrasound. Competence entails good hand-eye coordination for adequate image acquisition and appropriate anatomical interpretation [9]. The operator must acquire and interpret the image to visualize the needle and guide it to the desired target. However, the levels of competency may vary, with anesthesiology trainees possessing differing degrees of hand-eye coordination [9]. Structured teaching programs could help bridge the gap between trainees, ensuring high and equivalent levels of competence across healthcare services. Regional anesthesia trainees must acquire knowledge about the specific blocks prior to learning the basic practical skills [10]. This includes indications, relevant anatomy, complications, pre-procedure workup, and post-procedure management [10].

The lack of appropriate ultrasound equipment is a barrier to learning ultrasound-guided regional anesthesia [11]. In addition, trainees may not find enough time to incorporate regional anesthesia rotations due to time constraints [9]. Important factors facilitating learning ultrasound-guided nerve blocks include access to a formal structured training program and frequent exposure to learning opportunities [12]. The use of simulation training could also help achieve competency [13]. The implementation of such training programs carries extra costs and training times. A cost-benefit analysis should be performed by institutions/health services that decide to train anesthetists in ultrasound-guided peripheral nerve blocks. Ultrasound guidance could lead to reduced complications. It allows for a noninvasive method to visualize underlying structures, reducing the risk of puncturing them [6]. Increased patient safety could outweigh the costs associated with ultrasound training. Further research should compare complication rates between the LORT and the SIA to determine whether an investment in ultrasound training for FIB is justified.

The LORT is a viable option for developing countries that have limited access to ultrasound guidance equipment [14]. In addition, administering the LORT does not require ultrasound training, which may reduce costs associated with establishing robust and standardized treatment programs.

The reliability of our study's conclusions is subject to its limitations. Firstly, the results are based on a small sample size of seven cadavers, which limits their generalizability. Secondly, the integrity of anatomical structures in a cadaver and in vivo is different. The pattern of dye spread and nerves stained may differ from what would be observed in a patient. This was mitigated by the use of fresh frozen cadavers (as opposed to embalmed cadavers). Thirdly, both techniques were administered with cadavers lying supine, as opposed to the dorsal recumbent position used in clinical practice. The difference in positioning could have altered the spread of dye. Finally, it is possible that tissue disruption due to dissection resulted in a larger spread of dye had this not taken place. Further cadaveric studies with large sample sizes are required to more reliably compare both techniques. The method outlined in this work can be reproduced in subsequent research to collect further data. In addition, in vivo studies of large sample sizes comparing the effectiveness of both techniques can help determine which approach yields better analgesic outcomes. The effectiveness of different anesthetic medications should be compared to determine which one should be administered during FIB.

## Conclusions

The SIA and the LORT are used in clinical practice to provide analgesia in neck of femur fracture patients. A comparison of these techniques in seven cadavers revealed no statistically significant difference in the area of dye spread. The number of nerves stained was greater following the SIA. This suggests that the SIA may lead to superior anesthetic outcomes. The applicability of this conclusion is hindered by the use of cadavers and the small sample size in this study. Further cadaveric and in vivo studies comparing both techniques are required to determine which technique leads to better outcomes. The choice of technique is likely to be influenced by equipment availability and access to structured training programs for nerve blocks under ultrasound.
